# Genome-wide comparative analysis of metacaspases in unicellular and filamentous cyanobacteria

**DOI:** 10.1186/1471-2164-11-198

**Published:** 2010-03-25

**Authors:** Qiao Jiang, Song Qin, Qing-yu Wu

**Affiliations:** 1 School of Life Sciences, Tsinghua University, Beijing, 100084, China; 2 Yantai Institute of Coastal Zone Research, Chinese Academy of Sciences, Yantai, 264003, China

## Abstract

**Background:**

Cyanobacteria are an ancient group of photoautotrophic prokaryotes with wide variations in genome size and ecological habitat. Metacaspases (MCAs) are cysteine proteinases that have sequence homology to caspases and play essential roles in programmed cell death (PCD). MCAs have been identified in several prokaryotes, fungi and plants; however, knowledge about cyanobacterial metacaspases still remains obscure. With the availability of sequenced genomes of 33 cyanobacteria, we perform a comparative analysis of metacaspases and explore their distribution, domain structure and evolution.

**Results:**

A total of 58 putative MCAs were identified, which are abundant in filamentous diazotrophic cyanobacteria and *Acaryochloris marina *MBIC 11017 and absent in all *Prochlorococcus *and marine *Synechococcus *strains, except *Synechococcus *sp. PCC 7002. The Cys-His dyad of caspase superfamily is conserved, while mutations (Tyr in place of His and Ser/Asn/Gln/Gly instead of Cys) are also detected in some cyanobacteria. MCAs can be classified into two major families (α and β) based on the additional domain structure. Ten types and a total of 276 additional domains were identified, most of which involves in signal transduction. Apoptotic related NACHT domain was also found in two cyanobacterial MCAs. Phylogenetic tree of MCA catalytic P20 domains coincides well with the domain structure and the phylogenies based on 16s rRNA.

**Conclusions:**

The existence and quantity of MCA genes in unicellular and filamentous cyanobacteria are a function of the genome size and ecological habitat. MCAs of family α and β seem to evolve separately and the recruitment of WD40 additional domain occurs later than the divergence of the two families. In this study, a general framework of sequence-structure-function connections for the metacaspases has been revealed, which may provide new targets for function investigation.

## Background

Cyanobacteria are among the earliest branching groups on earth, dating back 2.5-3.5 billion years, based on the fossil evidence [1].As a taxonomic unit characterized by the ability to execute oxygenic photosynthesis, cyanobacteria constitute a group of species diverse in genome size and ecological habitats, indicating the significance of comparative genome research. Cyanobacteria, with a variation in genome size from 1.6 Mb (*Prochlorococcus *sp. MIT9301) to 9.2 Mb (*Nostoc punctiforme *PCC 73102), are found in almost every imaginable environment, from ocean to fresh water to bare rock. Cyanobacteria also inhabit in the extreme environments, for example, *Synechococcus *sp. JA-2-3B'a (2-13) and *Synechococcus *sp. JA-3-3Ab were separated from hot spring. As unicellular and non-nitrogen-fixing cyanobacteria, *Prochlorococcus *sp. and *Synechococcus *sp. maintain the smallest genome sizes and account for significant biomass and primary production of marine biosphere [2]. Other unicellular species have larger genome sizes, including water bloom forming cyanobacteria (*Synechocystis *sp. PCC 6803 and *Microcystis aeruginosa *NIES-843), a thylakoids absence cyanobacterium (*Gloeobacter *sp. PCC 7421), a nitrogen-fixing cyanobacterium (*Cyanothece *sp. ATCC 51142), and an animal-cyanobacterial symbionsis (*Acaryochloris marina *MBIC11017). While the diazotrophic filamentous cyanobacteria comprise the largest genome size, such as *Nostoc *sp. PCC 7120, *Anabaena variabilis *ATCC 29413, plant-cyanobacteria symbionsis *Nostoc *punctiforme PCC 73102 and marine *Trichodesmium *sp. IMS 101.

Programmed cell death (PCD) is a suicide mechanism to promote and maintain genetic stability [3]. PCD was considered as a characteristic of metazoans for a long time before apoptosis markers were found in yeast which indicates multicellularity is not the most important prerequisite[4]. Recently, PCD mechanism has been observed in all but one of the six/eight major groups of prokaryotes, with the exception of the rhizaria [5]. Experimental evidences for PCD in cyanobacteria come from three species, including the freshwater cyanobacterium *Anabaena *spp. exposed to univalent-cation salts, the bloom-causing cyanobacterium *Microcystis aeruginosa *from St. Lucie Estuary by treatment with H_2_O_2 _and *Trichodesmium *sp. IMS 101 suffering iron starvation and light irradiance [6-8].

Caspases (**c**ysteinyl **a**spartate-**s**pecific **p**rote**ases**) are one of the most important and widely researched apoptotic proteins in mammalian PCD. Caspase was initially thought to be limited to metazoans, and no one had managed to identify caspase homologues, either in plants or bacteria. Then Uren and his colleagues identified two ancient families of caspase-like proteins, paracaspases and metacaspases in silico [9] and Khan and his co-workers demonstrated that a yeast metacaspase (YCA1) mediates PCD in *Saccharomyces cerevisiae*[10]. Hereafter, metacaspases were found involved in PCD of yeasts, filamentous fungi, plants, and a variety of bacteria. Most of these metacaspases share sequence homology with caspases, but show different substrate specificity [11-16]. Metacaspases belong to caspase family (C14), which are part of the clan CD, a family of proteases characteristic with their His/Cys catalytic dyad [17]. Metacaspases process a conserved caspase catalytic subunit P20 domain (COG 4249, KOG1546 in the NCBI Conserved Domain Database), and share conserved amino acid residues within His- and Cys-catalytic sites [18]. Interestingly, most of the typical genes encoding in metazoan PCD are missing in bacteria and early-branching eukaryotes, such as CAD and P53[19]. Therefore, the presence of metacaspases suggests a concernful role within PCD evolution.

Cyanobacteria maintain a rich metacaspase pool, and many of these genes have been identified in silico [20] was identified in some sequenced cyanobacteria strains, including *Gloeobacter violaceus *PCC 7421, *Thermosynechococcus elongatus *BP-1, *Synechocystis *sp. PCC 6803, *Trichodesmium erythraeum *ISM 101, *Nostoc punctiforme *PCC 73102, *Nostoc *sp. PCC 7120, and *Anabaena variabilis *ATCC 29413. Metacaspases were absent in MED3, *Prochlorococcus marinus *MIT 9313, SS 120 (CCMP 1375), *Synechococcus *sp. WH 8102, *Synechococcus elongatus *PCC 7942 and *Synechococcus elongates *[20]. With the completion of genome sequencing of several cyanobacterial species, modifications and supplements are needed.

As of November 2008, 33 genomes of unicellular and filamentous cyanobacteria became available, which facilitates cyanobacterial systemic analysis for restriction-modification systems and serine/threonine protein kinases [21]. The comparative genome research on metacaspases and other PCD proteins in filamentous fungi has been documented [22]. Besides, Koonin and Aravind described a clear affinity of bacterial metacaspases and the metazoan caspases by phylogenetic analysis of caspase-like protease superfamily [19]. In this study, we selected five proven metacaspases in marine diatom *Thalassiosira pseudonana *to search for cyanobacterial metacaspases [11]. Metacaspases in *Thalassiosira pseudonana *were chosen due to the few metacaspases verified experimentally in cyanobacteria and the close evolutional relationship between cyanobacteria and eukaryotic phytoplankton. We employed a BLASTp-plus-phylogeny reconstruction approach [22] to analyze metacaspase sequences in cyanobacteria, and present an overall view of their classifications, structure, phylogeny and evolution. Better understanding of cyanobacterial metacaspases may provide further insights into evolution of PCD.

## Results

### Identification of metacaspase proteins

**Figure 1:**
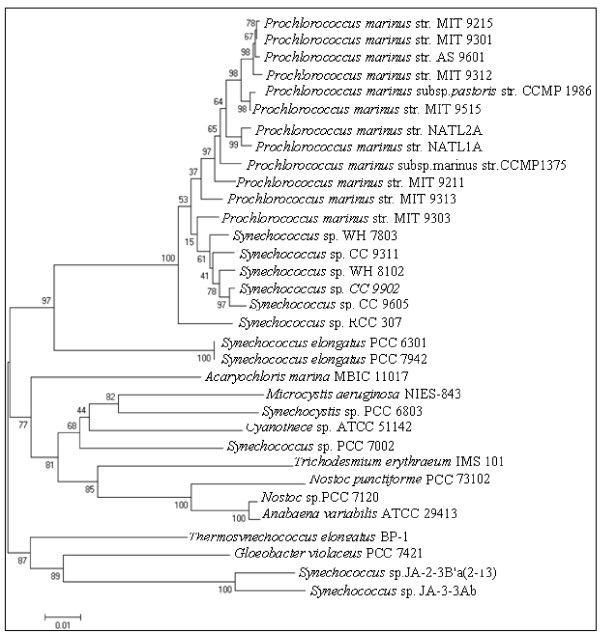
**Phylogenetic tree of the sequenced cyanobacterial strains based on 16s rRNA**. Phylogenetic tree reconstruction of the 33 fully sequenced cyanobacteria was performed based on 16s rRNA as described in the Methods section. The number on each branch indicates a bootstrap probability (1000 replicates).

The 33 complete cyanobacterial genomes downloaded from IMG database were used in this research (Table 1, Figure 1). To identify proteins similar to proven metacaspases from *Thalassiosira pseudonana*, we performed BLASTp searches of the 33 cyanobacterial genomes. CDD [23,24] and SMART [25] analyses with the derived sequences were then carried out to eliminate false positives. Two proteins annotated as caspase catalytic subunit P20 in IMG database, including 638107126 (IMG Gene Object Identifier) from *Trichodesmium erythraeum *ISM 101 and 641540115 from *Microcystis aeruginosa *NIES-843, were found to lack catalytic domain and excluded. Of two proteins (637313946 from *Thermosynechococcus elongatus *BP-1, and 638107265 from *Trichodesmium erythraeum *ISM 101), CASc domain (caspase catalytic subunit P20) recognized by CDD was not identified in SMART analysis [23], and both of the proteins were excluded. Altogether, 58 putative metacaspase sequences were considered in this study (Table 2). Twenty-six of which were originally annotated as peptidase C14, caspase catalytic subunit p20 or protein containing caspase domain. The remaining 32 proteins were accepted as metacaspases in this research, including 18 proteins annotated by other additional domains (such as ATPase, GUN4-like family protein, Chase2 sensor protein and WD40 repeats), 12 proteins annotated as hypothetical proteins, and 2 proteins annotated as unknown function protein DUF323.

**Table 1:** Sequenced cyanobacterial strains and MCA information

Strain	Key Feature	Total Proteins	Total MCAs&Percentage	H/C Sites	Additional Domains
*Prochlorococcus marinus *str. MIT 9215	Unicellular Marine	1983	-	-	-
*Prochlorococcus marinus *str. MIT 9301	Unicellular Marine	1907	-	-	-
*Prochlorococcus marinus *str. AS 9601	Unicellular Marine	1921	-	-	-
*Prochlorococcus marinus *str. MIT 9312	Unicellular Marine	1810	-	-	-
*Prochlorococcus marinus *subsp. *pastoris *str. CCMP 1986	Unicellular Marine	1717	-	-	-
*Prochlorococcus marinus *str. MIT 9515	Unicellular Marine	1906	-	-	-
*Prochlorococcus marinus *str. NATL2A	Unicellular Marine	2163	-	-	-
*Prochlorococcus marinus *str. NATL1A	Unicellular Marine	2193	-	-	-
*Prochlorococcus marinus subsp. marinus *str. CCMP1375	Unicellular Marine	1883	-	-	-
*Prochlorococcus marinus *str. MIT 9211	Unicellular Marine	1855	-	-	-
*Prochlorococcus marinus *str. MIT 9313	Unicellular Marine	2269	-	-	-
*Prochlorococcus marinus *str. MIT 9303	Unicellular Marine	2997	-	-	-
*Synechococcus *sp. WH 7803	Unicellular Marine	2533	-	-	-
*Synechococcus *sp. CC 9311	Unicellular Marine	2892	-	-	-
*Synechococcus *sp. WH 8102	Unicellular Marine	2519	-	-	-
*Synechococcus *sp. CC 9902	Unicellular Marine	2307	-	-	-
*Synechococcus *sp. CC 9605	Unicellular Marine	2645	-	-	-
*Synechococcus *sp. RCC 307	Unicellular Marine	2535	-	-	-
*Synechococcus elongatus *PCC 6301	Unicellular Freshwater	2527	1 (0.04%)	1	-
*Synechococcus elongatus *PCC 7942	Unicellular Freshwater	2612	1(0.04%)	1	-
*Acaryochloris marina *MBIC 11017	Unicellular Symbiont	6254	8(0.13%)	1	29
*Microcystis aeruginosa *NIES-843	Unicellular Freshwater	6312	2(0.03%)	1	-
*Synechocystis *sp. PCC 6803	Unicellular Freshwater	3172	1(0.03%)	1	-
*Cyanothece *sp. ATCC 51142	Unicellular Marine	4762	3(0.06%)	2	16
*Synechococcus *sp. PCC 7002	Unicellular Marine	2823	1(0.04%)	1	-
*Trichodesmium erythraeum *IMS 101	Filamentous Nonheterocystous	4451	10(0.22%)	1	62
*Nostoc punctiforme *PCC 73102	Filamentous Heterocystous Symbiotic	6087	9(0.15%)	2	6
*Nostoc *sp. PCC 7120	Filamentous Heterocystous Freshwater	5366	7(0.13%)	2	43
*Anabaena variabilis *ATCC 29413	Filamentous Heterocystous Soil	5043	9(0.18%)	2	64
*Gloeobacter violaceus *PCC 7421	Unicellular Rock	4430	4(0.09%)	-	56
*Synechococcus *sp. JA-2-3B'a(2-13)	Unicellular Hot spring	2862	1(0.03%)	1	-
*Synechococcus *sp. JA-3-3Ab	Unicellular Hot spring	2760	1(0.04%)	1	-

**Table 2:** Cyanobacterial putative metacaspase gene

Gene^a^	Family	His/Cys Sites	Additional Domians	Annotation
***Acaryochloris marina *MBIC 11017**				
641253645	cbMC α-other	H-Y C-G		Protease (caspase) p20 domain containing protein
641252580	cbMCβ		NACHT, WD40(13)	WD-40 repeat protein
641249149	cbMCβ		WD40(7)	WD-repeat protein
641257459	cbMCβ		Pentapeptide(4)	Peptidase C14, caspase catalytic subunit p20
641250651	cbMCβ		GUN4	GUN4-like family protein
641254504	cbMCα-TM			WD-40 repeat protein
641249463	cbMCβ		DUF323	Hypothetical protein
641257535	cbMCβ		DEXDc, HELICc	Dead/death box helicase domain protein
***Anabaena variabilis *ATCC 29413**				
637717727	cbMCα-other	H-Y C-S		Peptidase C14, caspase catalytic subunit p20
637718597	cbMCα-other			Peptidase C14, caspase catalytic subunit p20
637715366	cbMCβ		WD40(14)	Peptidase C14, caspase catalytic subunit p20
637719457	cbMCα-other			Peptidase C14, caspase catalytic subunit p20
637717526	cbMCβ		WD40(14)	Peptidase C14, caspase catalytic subunit p20
637717527	cbMCβ		WD40(8)	Peptidase C14, caspase catalytic subunit p20
637718424	cbMCβ		WD40(14)	Peptidase C14, caspase catalytic subunit p20
637718423	cbMCβ		WD40(14)	Peptidase C14, caspase catalytic subunit p20
637718962	cbMCα-TM	H-Y C-N		Peptidase C14, caspase catalytic subunit p20
***Cyanothece *sp. ATTC 51142**				
641679166	cbMCα-other	H-Y C-S		Putative peptidase C14, caspase catalytic
641676675	cbMCβ		NACHT, WD40(15)	WD-40 repeat protein
641678142	cbMCα-other	H-Y C-Q		Putative peptidase C14, caspase catalytic
***Gloeobacter violaceus *PCC 7421**				
637459639	cbMCβ		WD40(14)	WD-repeat protein
637458101	cbMCβ		WD40(14)	WD-40 repeat protein
637459020	cbMCβ		WD40(14)	WD-40 repeat protein
637461074	cbMCβ		WD40(14)	WD-40 repeat protein
***Microcystis aeruginosa *NIES-843**				
641536480	cbMCα-other			Peptidase C14, caspase catalytic subunit p20
641535722	cbMCα-other	H-Y C-S		Peptidase C14, caspase catalytic subunit p20
***Nostoc punctiforme *PCC 73102**				
638389336	cbMCα-other			Hypothetical protein
638391264	cbMCα-other			Hypothetical protein
638390474	cbMCα-other	H-Y C-S		Hypothetical protein
638392408	cbMCα-TM			Hypothetical protein
638386606	cbMCβ		ANF-receptor	ABC-type branched-chain amino acid transport systems, periplasmic component
638389051	cbMCβ		DUF323	Chromosome segregation ATPases
638388760	cbMCβ		Pentapeptide(3)	Uncharacterized protein containing caspase domain
638390427	cbMCα-TM	H-Y C-N		Uncharacterized protein containing caspase domain
638392333	cbMCβ		GUN4	Uncharacterized protein containing caspase domain
***Nostoc *sp. PCC 7120**				
637235546	cbMCα-other	H-Y C-S		Hypothetical protein
637230642	cbMCβ		WD40(14)	WD-40 repeat protein
637230643	cbMCβ		WD40(14)	WD-40 repeat protein
637232504	cbMCβ		WD40(14)	WD-40 repeat protein
637235425	cbMCα			Hypothetical protein
637233614	cbMCβ		DUF323	Hypothetical protein
637234068	cbMCα-TM	H-Y C-N		Hypothetical protein
***Synechococcus elongatus *PCC 6301**				
637616879	cbMCα-other	C-G		Hypothetical protein
***Synechococcus elongatus *PCC 7942**				
637798702	cbMCα-other	C-G		Hypothetical protein
***Synechococcus *sp. JA-2-3B'a(2-13)**				
637875026	cbMCα-other	H-Y C-G		Peptidase, C14 family
***Synechococcus *sp. JA-3-3Ab**				
637872245	cbMCα-other	H-Y C-G		ICE-like protease (caspase) p20 domain protein
***Synechococcus *sp. PCC 7002**				
641610111	cbMCα-other	H-Y C-S		ICE-like protease (caspase) p20 domain protein
***Synechocystis *sp. PCC 6803**				
637011435	cbMCα-other	H-Y C-G		Hypothetical protein
***Trichodesmium erythraeum *IMS 101**				
638107693	cbMCα-other	H-Y C-S		Peptidase C14, caspase catalytic subunit p20
638106962	cbMCβ		EZ-HEAT(15)	Peptidase C14, caspase catalytic subunit p20
638107555	cbMCβ		WD40(14)	Peptidase C14, caspase catalytic subunit p20
638109494	cbMCβ		WD40(15)	WD-40 repeat
638108799	cbMCβ		WD40(15)	WD-40 repeat
638107819	cbMCα-C			Peptidase C14, caspase catalytic subunit p20
638107188	cbMCβ		DUF323	Protein of unknown function DUF 323
638107169	cbMCα-other			Peptidase C14, caspase catalytic subunit p20
638107752	cbMCβ		DUF323	Protein of unknown function DUF 323
638105709	cbMCβ		CHASE2	Putative Chase2 sensor protein

a: the numbers indicate "IMG Gene Object Identifier" in IMG database

Amid diverse cyanobacterial genomes, the number of metacaspase genes varies from 0 to 10. Within unicellular cyanobacteria, Symbiont *Acaryochloris marina *MBIC 11017 has 8 MCAs, much more than other species. Correspondingly, the percentage of metacaspases within total proteins (0.13%) is highest among unicellular cyanobacteria. All of the marine *Synechococcus *strains lack MCAs except for *Synechococcus *sp. PCC 7002. Only one metacaspase gene was found in the *Synechocystis *sp. PCC 6803. Four *Synechococcus *strains inhabit in freshwater, land and hot spring contain one metacaspase for each, including PCC 6301, PCC 7942, JA-2-3B'a(2-3), and JA-3-3Ab. Water-blooming cyanobacterium *Microcystis aeruginosa *NIES-843 contains two MCAs. The percentages of MCAs within total proteins (0.03% -0.04%) in *Synechocystis *sp., *Synechococcus *sp. and *Microcystis *sp. strains are the lowest. The other two unicellular cyanobacterial strains, *Gloeobacter violaceus *PCC 7421 and *Cyanothece *sp. ATCC 51142, maintain 3 to 4 MCAs and have moderate percentages of MCAs in total proteins: 0.09% and 0.06%, respectively.

Compared to unicellular cyanobacteria, filamentous diazotrophic cyanobacteria have more metacaspase genes (10 for *Trichodesmium erythraeum *IMS 101, 9 for *Anabaena variabilis *ATCC 29413, 9 for *Nostoc punctiforme *PCC 73102, and 7 for *Nostoc *sp. PCC 7120). Among the filamentous cyanobacteria, *Nostoc punctiforme *PCC 73102 has the largest genome; however, nonheterocystous cyanobacterium *Trichodesmium erythraeum *IMS 101 contains the largest number of metacaspases and the highest percentage in total protein (0.22%) (Table 1).

One or two MCAs containing mutations of conserved catalytic sites (His/Cys) were found in every cyanobacterial strain, exclusive of *Gloeobacter violaceus *PCC 7421. In most cases, the His residues are replaced by Tyr, and the Cys residues mutate into Ser, Asn or Gly. Uniquely, within the metacaspase (641678142) from *Cyanothece *sp. ATTC 51142, Gln substitutes for the Cys residue (Table 1, 2).

### Structure and function

**Figure 2:**
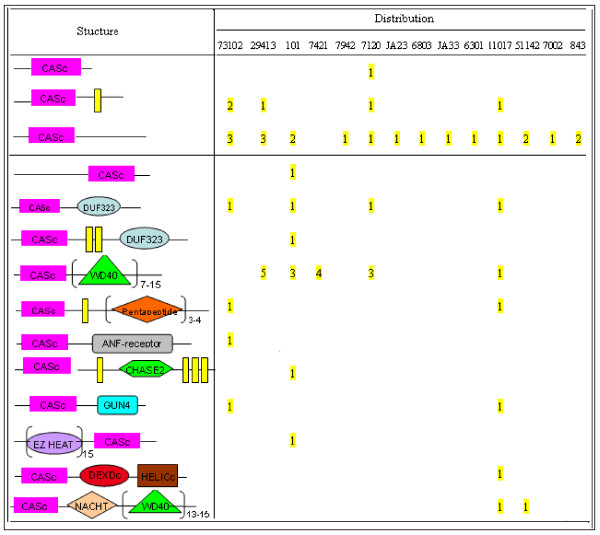
**Domain organization and distribution of putative cyanobacterial metacaspases**. Fused domains that form a single polypeptide chain are connected by a horizontal line. The yellow rectangles represent transmembrane (TM) domain. Strain and domain names are as in Table 1 and 3, respectively. Figures are not drawn to scale.

Based on structural characteristics, we classify the identified cyanobacterial metacaspases into two families, cbMC α and cbMC β (Figure 2).

Cyanobacterial metacaspase Family α (cbMC α) includes 27 MCAs that process none other identifiable domains than P20. This family was further divided into four subfamilies. Subfamily α (cbMC α-I) processing the CASc domain only, contains one MCA (637235425 from *Nostoc sp*. PCC 7120). Subfamily II (cbMC α-TM) maintaining a single transmembrane (TM) domain, contains five MCAs found in filamentous cyanobacteria *Anabaena variabilis *ATCC 29413, *Nostoc punctiforme *PCC 73102, *Nostoc *sp. PCC 7120, and unicellular cyanobacterium *Acaryochloris marina *MBIC 11017. Subfamily III (cbMC α-other) contains 20 proteins, which is the majority in cyanobacterial MCAs. These MCAs have an unidentified C-terminal domain each, and distribute among all cyanobacterial strains except *Gloeobacter violaceus *PCC 7421. MCAs attributed to subfamily I to III process CASc in their N-terminal. While the single MCA of Subfamily IV (cbMC α-C), 638107819 from *Trichodesmium erythraeum *ISM 101, holds the CASc domain in the C-terminal.

Cyanobacterial metacaspase Family β (cbMC β) occupying at least one additional domain, comprises 31 (53.4%) metacaspases from all filamentous cyanobacteria and three unicellular species (*Cyanothece *sp. ATCC 51142, *Acaryochloris marina *MBIC 11017, and *Gloeobacter violaceus *PCC 7421). The number of additional domains varies from 16 to 56. *Gloeobacter violaceus *PCC 7421 contains the largest number of additional domains, which is 14 times than that of the total MCAs (Table 1). Seven unicellular strains lack additional domains, including *Synechococcus *sp. strains, *Microcystis aeruginosa *NIES-843 and *Synechocystis *sp. PCC 6803.

In total, 10 types of additional domains were identified in cyanobacterial MCAs: ANF-receptor, WD40, GUN4, NACHT, DUF323, CHASE2, Pentapeptide, DEXDc, HELICc, and EZ-HEAT (Figure 2, Table 3). Most of these domains are involved in signal transduction, for example CHASE2, GUN4, ANF-receptor and WD40 (Table 3). In addition, prevalent domains with scaffolding or unknown functions in bacteria were identified, such as DUF323, EZ HEAT and Pentapeptide [21]. Two domains within helicases, HELICc and DEXDc, were found to fuse together in 641257535 of *Acaryochloris marina *MBIC 11017. Interestingly, PCD related domain NACHT was also detected in two MCAs (641676675 from *Cyanothece *sp. ATCC 51142 and 641252580 from *Acaryochloris marina *MBIC 11017).

**Table 3:** Additional domains of cyanobacterial MCA Gene

Abbreviation	Domain	Functions	Distribution
ANF-receptor	ANF-receptor	extracellular ligand binding domain	transmembrane receptors (such as histidine kinases, serine/threonine kinases)
CHASE2	CHASE2	extracellular sensory in signal transduction	transmembrane receptors (such as histidine kinases, serine/threonine kinases)
DEXDc	DEAD-like helicases superfamily	ATP-binding	proteins involved in ATP-dependent RNA or DNA unwinding
DUF323	Domain of unknown function	unknown	bacterial unknown proteins
EZ HEAT	E-Z type HEAT repeats	scaffold	cyanobacterial phycocyanin lyase and other proteins
GUN4	GUN4-like domain	signaling; accumulation of glycolipids into the heterocysts	cyanobacterial serine/threonine kinases
NACHT	NACHT domain	programmed cell death	apoptotic proteins
WD40	WD40 domain	regulator in signal transduction	cyanobacterial serine/threonine kinases
pentapeptide	pentapeptide repeats	accumulation of glycolipids into the heterocysts	unicellular and filamentous cyanobacterial proteins

WD40 repeats, the most prevalent additional domains identified in 6 cyanobacterial species, replicate for 7 to 15 copies in a single metacaspase protein. Some additional domains were identified exclusively in a particular metacaspase protein, including DEXDc and HELICc in 641257535 (*Acaryochloris marina *MBIC 11017); EZ HEAT in 638106962 (*Trichodesmium erythraeum *ISM 101), CHASE2 in 638105709 (*Trichodesmium erythraeum *ISM 101), and ANF-receptor in 638386606 (*Nostoc punctiforme *PCC 73102).

### Phylogenetic analysis

**Figure 3:**
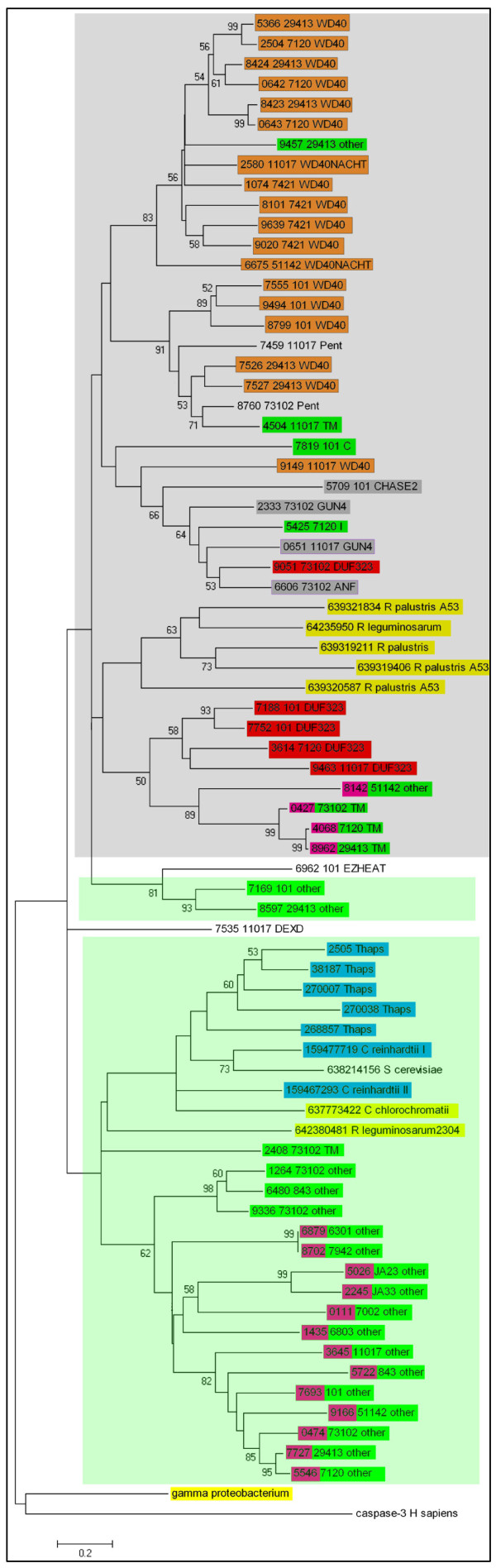
**Phylogenetic tree of the conserved catalytic domains of metacaspases**. The phylogenetic tree of metacaspase catalytic domains was constructed as described in Methods section. Bootstrap values >50% are indicated on the branches (1000 replicates). Cyanobacterial metacaspase gene IDs and the strain names are as in Table 2 and Table 1, separately. Strain names, families and additional domains are also given following the IDs. The green and grey boxes indicate Clade I and II, separately. MCAs containing the His/Cys mutation are marked in pink. MCAs from subfamily cbMC α are marked in green. Major additional domains from cbMC β are marked in color: orange-WD40, grey-signalling domains, and red-DUF323. MCAs from different species are marked in color: blue-eukaryotic plankton, yellow-eubacteria except cyanobacteria.

Considering the confusion created by additional domains with possibly separate evolutionary histories, the conserved catalytic domains of MCAs instead of their whole sequences were used during the phylogenetic study (Figure 3). The catalytic domains, about 340 amino acids in length, were identified using SMART and CDD databases [23,26]. The MCA phylogenetic tree was rooted in the human caspase-3 and the putative metacaspase of Gamma proteobacterium.

The phylogeny tree comprises two clades in general. MCAs from cbMC α belong to Clade I (green box in Figure 3) that also includes two bacterial MCAs (637773422 from *Chlorobium chlorochromatii *and 642380481 from *Rhizobium leguminosarum*) and seven eukaryotic MCAs from *T. pseudonana, S. cerevisiae *and *Chlamydomonas reinhardtii*. All of these MCAs are orthologues because of obvious evolutionary relationships with high bootstrap value. Clade II (grey box in Figure 3) contains members of cbMC β family with WD40, DUF323 and signalling domains, which cluster separately according to the additional domains. Those from subfamily cbMC α-TM and eubacteria (*Rhizobium leguminosarum *and *Rhodopseudomonas palustris*) attribute to Clade II as well. In a word, most photosynthetic bacterial MCAs cluster with MCAs of cyanobacterial family β and the eukaryotic MCAs gather with family α. Within each clade, the MCAs cluster according to the phylogeny of the species.

Within clade I, one copy of MCA containing the His/Cys mutation is found in each cyanobacterium that maintains MCA, except for *Gloeobacter violaceus *PCC 7421. All of these MCAs belong to cbMC α-other and cluster strictly based on the phylogeny of the species. All of the mutated MCAs in clade I are orthologs because of their close evolutionary relationships. Besides, four mutated MCAs from one unicellular and three filamentous cyanobacteria gather together in clade II.

*Anabaena variabilis *ATCC 29413 and *Nostoc *sp. PCC 7120, two filamentous species that show close evolutionary relationships in 16S rRNA tree, share 3 pairs of MCA sequences in clade I. Four WD40-containing MCAs from *Gloeobacter violaceus *PCC 7421 form a separate cluster together. In addition, some MCAs adjacent to each other on the chromosome display close evolutionary relationships, including 637717526/637717527, 637718423/637718424 of *Anabaena variabilis *ATCC 29413 and 637230642/637230643 of *Nostoc *sp. PCC 7120. Some MCAs of subfamily α are flanked with two WD40- or GUN4-containing MCAs.

## Discussion

Although metacaspases do not cleave caspase substrates [12,18,27-29], several evidences have been given to support their roles in PCD in plants (see review [30]). For example, when challenged by the plant pathogen, *Arabidopsis *KO lines of metacaspase suppress cell death [31]. *Arabidopsis *metacaspse-8 KO lines triggered by UVC or H_2_O_2 _display reduced cell death [12]. Moreover, in the suspensor cells of an embryogenic culture of *Picea abies*, down-regulation of MCA leads to a phenotype with a reduced cell death [32].

What makes metacaspases so interesting? First, most of the metazoan PCD-related genes are lost in unicellular organisms, excepting metacaspases that play vital roles in PCD of eukaryotic planktons and yeasts. Second, compared with caspases and paracaspases identified in higher animals, metacaspases are widespread among bacteria, fungi and plants, which suggest their early evolutionary positions [33].

Bidle and Falkowski identified cyanobacterial and phytoplankton metacaspases in silico, and explored the evolution deeply [20]. With the completion of genome sequencing of several cyanobacterial species, modifications and supplements are needed. For example, MCA was reported to be absent in *Synechococcus *sp. PCC 7942, but a protein (ID: 637798702, annotation: hypothetical protein) was found to contain P20 domain. Cyanobacterium *Thermosynechococcus elongatus *BP-1 was proved to have no metacaspase orthologue in our study. Moreover, with the release of genomic sequence, MCAs were identified in *Synechococcus *sp. JA-3-3Ab and *Synechococcus *sp. JA-2-3B'a(2-13).

The distribution of putative metacaspase encoding open reading frames (ORFs) in cyanobacteria is an integrated function of the genome sizes and the ecophysiological properties. Most cyanobacteria process proportionate numbers of putative metacaspase genes with genome sizes, except for symbiotic *Acaryochloris marina *MBIC 11017. Though death is not the only way to adapt to environmental changes, for example, cyanobacteria modify their metabolism in response to different stress conditions [34], death is still a direct and drastic cellular response to environmental changes. Thus diverse distributions of metacaspase genes may reflect various environmental selective pressures. For example, putative MCA encoding ORFs are not widespread through unicellular cyanobacteria (Table 1). All of the *Prochlorococcus *and *Synechococcus *strains lived in the oligotrophic open ocean lack putative metacaspase genes. While *Synechococcus *strains that inhabit in freshwater and hot spring still maintain one metacaspase encoding ORF. Considering the similar genome size, environmental selective pressure may take responsibility for this difference. Parallel conclusion was provided by Serine/threonine kinases in cyanobacteria indicating remarkable reduction of signal transduction proteins and environmental stress response systems in the ocean [21]. Gene lost is revealed to facilitate these cyanobacteria to acclimatize to the oligotrophic environment. The major driving force was supposed to be "a selective process favouring the adaptation of these cyanobacteria", which was discussed by Alexis Dufresne *et al*. in detail [35]. Filamentous heterocystous cyanobacteria, on the other hand, differentiate heterocysts in response to the absence of combined nitrogen, and exhibit ecological properties including broad symbiotic competence with plants and fungi [21], contain more putative MCA encoding ORFs even after allowing for their larger genome sizes [36].

The symbol of caspase superfamily is the possession of catalytic P20 domain and the conserved Cys-His dyad, forming the "specificity pocket" [9]. Within cyanobacterial metacaspases, sequence contexts of His and Cys are basically the same as those in caspases (His:**(Y/F)SGHG**, and Cys:**QAC(R/Q)G) **[17]. The maintaining of the conserved His and Cys indicate the importance of these catalytic sites. Interestingly, 17 cyanobacterial MCAs encode Tyr in place of His and Ser/Asn/Gln/Gly instead of Cys. Likewise, of two metacaspases in *T. brucei*, TbMCA1 and TbMCA4, Ser occupies the site of the putative Cys [37]. Considering the fact that experimental mutation of the active-site cysteine to serine resulted in inactive of some cysteine peptidases, mutated cyanobacterial metacaspases may be catalytically inactive.

Domain fusion provides a chance to recruit related functions in a single protein, especially within bacteria which maintain smaller genomes and compact gene clusters [19]. Additional domains of cyanobacterial MCAs, such as GUN4 [38] and WD40 [39], illuminate the signalling pathways involved in PCD. Owe to the considerably specific proteolytic activity and proximity-induced activation, the signalling domains may be the target of the metacaspases. Consequently, metacaspases may take a share in signalling mediation instead of mere protein degradation. Two domains within helicases, HELICc and DEXDc, were found to fuse together in 641257535 of *Acaryochloris marina *MBIC 11017, which implies the interactions between the caspase proteolytic activity and ATP-dependent RNA/DNA unwinding. In addition, domains with same functions tend to assemble in a protein [19], therefore the identification of NACHT [19,22,40] domain reinforces the possibility of metacaspase involving in PCD. Compared with metazoan caspases, bacterial metacaspases may present a minimal set of apoptotic machinery. Additional domains are typically employed as "sensor response modules" and form multi-domain proteins with MCAs to participate in signal transduction. It can be imagined that caspases recruit additional domains or even large motifs to apoptotic complexes in the evolution.

Previous classification criterion of MCAs is based on the prodomain, and MCAs can be classified into two families ("Type I with an N-terminal extension" and "Type II with a linker region between the putative large and small subunits") [9,18,36]. However, most cyanobacterial MCAs maintain a C-terminal extension without the linker region and have varied additional domains (Figure 2, Figure 3). Therefore a novel categorization standard based on the additional domain was given in this research. To avoid future confusion of the MCA families, the names "Type α" and "Type β" were used instead of "Type I" and "Type II".

The obtained metacaspase phylogenetic tree indicates that MCAs display clear-cut relationships, based strictly on structural characteristics and the phylogeny of the species. Moreover, compared to the species phylogeny, structural characteristics play a more critical role. The tree of catalytic P20 domains coincides well with the phylogenies based on 16s rRNA, indicating the rare gene gain-and-loss events and the importance of MCAs that remain conserved in history. Besides, four WD40-containing MCAs from *Gloeobacter violaceus *PCC 7421 form a separate cluster indicating obvious lineage-specific duplication events. *Anabaena variabilis *ATCC 29413 and *Nostoc *sp. PCC 7120, two filamentous species that enjoy a very close evolutionary relatedness in 16S rRNA tree, share 3 pairs of MCA sequences in clade I. These nonorthologous MCAs may be produced by gene duplication before the divergence of the two species.

Most photosynthetic bacterial MCAs cluster with MCAs of cyanobacterial family β (with additional domain) and the eukaryotic MCAs cluster with family α (without additional domain). The presence of the mutated MCAs and their convergences suggest that MCAs of family α and β evolve separately. One possibility is that the recruitment of WD40 additional domain occurs later than the divergence of the two clades.

## Conclusions

The availability of cyanobacterial genome sequences facilitates comparative analysis. Metacaspases, sequence homologs to caspases, play key roles in programmed cell death (PCD) in several prokaryotes, fungi and plants. Among 33 species of cyanobacteria, a total of 58 putative metacaspase genes have been identified. The quantity of metacaspase genes in unicellular and filamentous cyanobacteria depends on the genome size and ecological habitat. The Cys-His dyad of caspase superfamily is conserved in most cyanobacterial MCAs, however, Tyr and Ser/Asn/Gln/Gly residues have also been detected in the sites of His and Cys within some metacaspases. Ten types and a total of 276 additional domains were identified, most of which may involve in signal recognition. Programmed cell death related NACHT domain was also found in cyanobacterial metacaspases. Phylogenetic tree of MCA catalytic P20 domains coincides well with the phylogenies based on 16s rRNA.

## Methods

Thirty-three species of cyanobacteria, including *Prochlorococcus*, *Synechococcus*, *Synechocystis*, *Gloeobacter*, *Cyanothece*, *Microcystis*, *Trichodesmium*, *Acaryochloris*, *Anabaena *and *Nostoc *were used in this analysis. Since sequences of 36 species had not been fully released, they were not considered in our comparisons. All of the 33 genome sequences (as of Nov. 2008) were accessed from IMG in FASTA format [41].

In order to identify genes that may encode metacaspases, proven metacaspases from marine diatom *Thalassiosira pseudonana *(Protein id: 270038, 2505, 268857, 270007, 38187 in *Thalassiosira pseudonana *"finished chromosomes" database v3.0 [11,42]) were used to construct a query protein set. BLASTp (protein-protein BLAST) [22,43,44] was conducted locally to search all proteins from each of the 33 cyanobacteria. Proteins found by this method that fit the criteria for a genuine metacaspase were added to the query set for another round of BLASTp searches. A threshold e-value of 1e-10 was set in the first two rounds, which changed into 2e-20 subsequently. The procedure was continued until no new proteins were found.

Proteins identified by BLASTp were aligned using Clustal X (Version 1.83) program [45] with a gap opening penalty of 10, a gap extension penalty of 0.2, and Gonnet as the weight matrix. The alignment was examined by inspection of peptidase C14, caspase catalytic subunit P20 domain (COG 4249, KOG1546 in the NCBI Conserved Domain Database [23,24]). A protein was accepted as a metacaspase if it was possible to recognize P20 domain and if the most conserved His and Cys residues known to participate in the function of metacaspases [18] were present. However, minor alterations of the conserved His and Cys residues were tolerated. Specifically, putative MCA genes encoding Tyr in place of His and Ser/Asn/Gln/Gly instead of Cys were taken into account as well. Structure analyses of the obtained metacaspases were performed using the SMART (**S**imple **M**odular **A**rchitecture **R**esearch **T**ool) [25,26] and the CDD (**C**onserved **D**omains **D**atabase) [23,24], relying on hidden Markov models and Reverse Position-Specific BLAST separately. Sequences of the P20 domain (about 300 aa in length) used for phylogenetic tree construction were obtained from the SMART database [25,26]. Trees based on metacaspase P20 domain and cyanobacterial 16s rRNA were constructed using NJ methods of the MEGA package (Version 4.0) [46], and the reliability of each branch was tested by 1000 bootstrap replications. In phylogenetic analysis of MCA, putative metacaspase of Gamma proteobacterium and human caspase-3 were used as outgroups to root the tree.

## Authors' contributions

Qiao Jiang conceived of the study, participated in the sequence analysis, and drafted the manuscript. Song Qin and Qing-yu Wu participated in its coordination and helped to draft the manuscript. All authors read and approved the final manuscript.
